# Novel soil-less potting mixes for the mycorrhization of *Quercus pubescens* Willd. seedlings with *Tuber melanosporum* Vittad.

**DOI:** 10.1186/s12870-023-04194-y

**Published:** 2023-05-12

**Authors:** Sofia Pagliarani, Andrea Vannini, Elena Kuzminsky, Carmen Morales-Rodríguez

**Affiliations:** Department of Innovation of Biological Systems, Food and Forestry DIBAF, Tuscia University, Viterbo, Italy

**Keywords:** Downy oak, Mycorrhization improvement, Compost-based substrates, Périgord black truffle, Root system growth, Boron

## Abstract

Truffle cultivation has quickly grown in Europe and elsewhere as a consequence of the increase in the demand of the market. Thus the optimization of the protocols for the production of elite mycorrhized plants are also needed, keeping in consideration the economic and environmental sustainability. The suitability of two compost-based potting mixes to produce *Quercus pubescens* Willd. plants mycorrhized with the black Périgord truffle *T. melanosporum* Vittad. was tested as an alternative to the traditional potting mix used. The effects on mycorrhizal development and the morphometric assessment of the root and shoot system of the *Q. pubescens* seedlings were investigated eight months after the spore slurry inoculation in a glasshouse experiment. From the results obtained, the compost mix containing green organic residues from pruning and mowing (Mix 2) achieved better performance than the control and the potting mix based on composted municipal organic wastes, showing significantly higher mycorrhization percentage, root length, number of root tips, and root forks. In conclusion, a potting mix containing recycled green organic matter, which is readily available, cheap, and environmentally sustainable, can offer excellent mycorrhization performances and may be included in the mycorrhization process of downy oak seedlings with *T. melanosporum* under controlled conditions.

**Supplementary Information:**

The online version contains supplementary material available at 10.1186/s12870-023-04194-y.

## Introduction

Woody plants and soilborne fungi form a mutualistic interaction known as ectomycorrhizal (ECM) symbiosis [1]. *Tuber* spp. are hypogeal ascomycetes that need to form a symbiotic relationship with host plants to propagate vegetatively and form truffle fruiting bodies. Broadleaved forests and the plantations in the Mediterranean basin in Italy, France, and Spain represent the most productive environments for the production of the Périgord black truffle, *Tuber melanosporum* Vittad. [2–5]. Due to the organoleptic qualities of its fruitbodies, *T. melanosporum* truffles represents one of the most demanded *Tuber* species in the world [6] and one of the most relevant in terms of global consumption and economic importance [7, 8]. In the second half of the 20^th ^century, intensive orchard farming started [9] due also to the introduction of protocols to stimulate *T. melanosporum* ectomycorrhizal production in controlled conditions [10]. The actual global yield is approximately 120.000 kg per year, which generates yearly revenue of approximately € 50 million for truffle farmers [11, 12] with retail prices of ca. 700 − 1500 euros per kilogram based on season and meteorological variables [3, 8, 13]. In France, Italy, and Spain, productive truffle orchards provide rural landowners with an alternative to agricultural subsidies, encourage the regeneration of abandoned crop fields, and require little agricultural inputs [14]. Nowadays, these delicacies are grown all over the world by cultivating truffières mostly associated with European *Quercus* seedlings [2, 3, 15, 16]. Oak species are considered long-time producers, extending their truffles production far longer than other host symbionts such as *Corylus avellana* L. [17]. The ability to produce ECM plants under controlled conditions for truffle cultivation is now regarded as the most significant development in truffle cultivation science. If done properly, it allows the formation of ECM seedlings free of competitors and contaminating species. Using this technique, numerous plantations of inoculated trees have been developed outside the natural range of *T. melanosporum*, that is in Australia and New Zealand [18]. Even though numerous independent research efforts have been devoted to ameliorating *T. melanosporum* inoculation procedures [19], often production protocols remain a trade secret [7]. Watering, fertilization, temperature, light levels, and potting medium formulation are important factors to optimize the production of ectomycorrhizal seedlings [20]. However, considering the production’s environmental and economic sustainability, the composition of the potting medium can be considered a critical element. Indeed, peat is the most used substrate for plant production in nurseries [21] due to its optimal physical, chemical, and biological properties for plant growth and development [22], and its high availability in the northern hemisphere, making it a relatively inexpensive material [23]. However, peat is a limited resource, and overuse will deplete supplies and have significant environmental consequences [24]. Peat-exporting countries in northern and central Europe have curtailed peat extraction [25]. Several studies have been conducted to identify alternative, effective, and sustainable growing substrate components for soilless agriculture [26–28]. Compost can be employed in numerous applications, including bio fertilization and soil enhancement, peat replacement in growing substrates [29–31], carbon sequestration, maintaining or increasing soil organic matter, and reducing greenhouse gas emissions [32]. Decomposition processes are part of a sustainable waste management approach that is nearly in line with the zero-waste concept: encouraging the conservation and redesign of resource life cycles for reuse in other processes [33]. Compostable substrates derived from the decomposition of organic waste (green and municipal organic residues) could serve as a viable alternative. Composting is the process of biologically decomposing organic waste under aerobic conditions [34]. The compost produced from biowaste can be reused as nutrient-rich fertilizers or growing substrates [35, 36]. Therefore, this study aims to investigate compost-based potting mixes as substrates for the artificial mycorrhization of *Q. pubescens* Willd. seedlings with *T. melanosporum*, specifically investigating the quality and quantity of mycorrhization, associated with root parameters and overall plant development. The final objective of the present study is to offer an alternative to truffle potting mixes used for mycorrhization in the general context of circular economy and sustainable use of natural resources.

## Material and methods

### Soil-less potting mix to produce high-quality mycorrhizal plants

#### Plant material

Truffleland Ltd (Scheggino, Italy) supplied *Q. pubescens* acorns, which were surface sterilized following the protocol of Benucci et al. [37]. After sterilization, the plant material was put in heated propagation trays containing sterile perlite until germination. A total of 180 germinated seedlings of *Q. pubescens* were transplanted in 9 × 9 × 13 cm-deep plastic pots (0.5 L, Bamaplast S.r.l.) containing the three different sterilized potting mixes (see Sect. Potting mixes for the composition). Experimental trials were conducted at Truffleland and consisted of 20 seedlings per potting mix replicated three times (60 seedlings per potting mix). The plants were arranged in randomized blocks for each treatment in greenhouse conditions with a mean daily air temperature varied between 20–25 °C, and relative humidity ranged from 50 to 70%. The plants were watered by a micro-sprinkler to reach field capacity with a frequency of irrigation depending on the time of year: every day from June to August and every 2 days from September to January.

#### Potting mixes

The two potting mixes employed were prepared using different organic wastes allowed in organic farming with chemical, and physical characteristics in agreement with the Italian Legislative Decree 75/2010. In detail, Mix 1 consisted of 50% sterilized mixed composted amendment (municipal and green organic residues) provided by a composting plant of the Acea group (https://www.gruppo.acea.it), supplemented with 35% of perlite, 15% of sand, and a slow-release fertilizer (Osmocote™, 2 kg/m^3^); Mix 2 was composed of 50% sterilized green composted amendment (green organic residues from pruning and mowing) provided by the company Mechelli Ltd (http://www.terriccimechelli.it/), and supplemented with 35% of perlite, 15% of sand, and Osmocote™ (2 kg/m^3^). The soil-based mycorrhization substrate used as ‘business as usual’ by Truffleland Ltd. was considered a control mix. Table 1 provides a summary of the physical and chemical properties of the three potting mixes. Analysis of the heavy metals contained in the two composts used (Mix 1 and Mix 2) such as Chrome (Cr), Cadmium (Cd), Mercury (Hg), Nickel (Ni), Lead (Pb), Zinc (Zn,) and Copper (Cu) are below the legal limits (Italian Legislative Decree 75/2010).

**Table 1:** Chemical-physical analysis of potting mixes: Mix 1: green compost from Acea group; Mix 2: green composts from Mechelli Company and the ‘business as a usual’ potting mix from Truffleland Ltd. as control. The three substrates were used for the mycorrhization of *Q. pubescens* seedlings with *T. melanosporum*

Property	Control	Mix 1	Mix 2
**Physical**			
pH, unit	7.7	7.1	7.4
Total limestone, %	41.6	16.6	19.0
Active limestone, %	12.1	1.7	1.2
Organic C, %	3.43	8.03	7.18
Organic matter, %	5.92	13.86	12.39
Sand, %	29	76	76
Silt, %	30	13	12
Clay, %	41	11	12
Texture	Clayey	Sandy loam	Sandy loam
**Chemical**			
N total, %	0.320	0.683	0.614
C/N	10.7	11.7	11.6
Calcium (Ca), ppm	5,220	5,940	6,300
Magnesium (Mg), ppm	154	602	645
Potassium (K), ppm	355	2,290	2,524
Sodium (Na), ppm	126	468	548
Boron (B), ppm	0.30	3.92	2.22
Phosphorus (P), ppm	10	110	92
Zinc (Zn), ppm	1.4	15.4	13.6

#### Fungal material

 In February 2019, mature ascomata of *T. melanosporum* provided by Truffleland Ltd (https://www.truffleland.com) were cleaned with a brush to remove soil, washed in water, and stored at -20 °C until use [38]. The truffles chosen for the inoculation were examined externally and microscopically to assess their identity [15, 37–39] and the presence of light brown to mid-brown colored mature spores [40].

In May 2019, a truffle spore-slurry was prepared by mixing frozen truffles with water. The spore’s density was counted with a Neubauer counting chamber (Marienfeld Superior, Germany) under a light microscope Axioskop 50 (Zeiss, Oberkochen, Germany) at 400 × magnification and brought to a final concentration of 3.5 × 10^4^ spores/ml. A rotating dispenser was used to distribute 1 g of spore-slurry to each seedling in the pots.

#### Assessment of percent of mycorrhization

To visualize ECMs, the root system was gently washed and visualized using the stereomicroscope (Visioscope ZTL350, VWR) [41]. ECMs colonization rate was estimated using the Relative Abundance Value (RAV) methodology described by Bencivenga et al. [42]. Briefly, two sectors of equal length were identified in the root system of each seedling, one proximal and one distal; four portions of roots were identified in both the proximal and distal part; from each portion 50 root apices were counted, separating them into 1) mycorrhized by *T. melanosporum*, 2) mycorrhized by other fungal species, 3) not mycorrhized. The average values of root mycorrhization were determined by counting the total number of mycorrhized tips with *T. melanosporum* expressed as a percentage over the total number of tips examined (400).

#### Molecular identification of Tuber melanosporum ECMs

Total DNA was extracted from 200 mycorrhized root tips per sample using the NucleoSpin Plant II kit (Macherey–Nagel; Düren, Germany). The primers TM1b and ITS4 [43] and the Go Taq Hot Start (Promega; Wisconsin, United States) were used for *T. melanosporum* amplification. Both positive and negative controls were used at each stage. Amplification was carried out following the protocol of Séjalon-Delmas et al. [43]. Amplicons were cleaned using the NucleoSpin Gel and PCR Clean-up Reagent kit (Macherey–Nagel; Düren, Germany), following the manufacturer's specifications. The cleansed products were loaded onto a 1.5% agarose gel to confirm the presence and size of the expected amplicon.

#### Morphometric assessment of plants

The shoot height and root collar diameter were measured immediately after the seedling’s inoculation and at the end of the experiment (8 minths). The WinRHIZO® software package (v. 5.0, Regent Instruments, Inc., Quebec, QC, Canada) was used to determine the total root length, root tips, and forks [44, 45]. Roots were first separated at the collar from the above-ground part of the seedlings and gently washed in running water to remove soil debris. The roots were then positioned in between Plexiglas trays and scanned using the Epson Perfection V700 Photo Scanner system (Epson America, Inc., 126 Long Beach, CA, USA) to generate a two-dimensional picture of each root. Afterwards, shoots and roots were oven-dried (80 °C for 2 weeks) and weighted [46].

#### Data analysis

Principal Component Analysis (PCA) was carried out with PAST 4.03 [47]. GraphPad Prism version 8.00 (GraphPad Software, San Diego, CA, USA) was used for parametric statistics. The Shapiro–Wilk test (= 0.05) was used to check for normal distribution of datasets. Datasets normalization was carried out where needed by data transformation. The homogeneity of variance was checked by Levene’s test. One-way ANOVA was employed to analyzed datasets. Comparisons of all possible groups pairing was carried out with the Tukey's post-hoc test.

## Results

### Mycorrhizal colonization 

#### Seedlings survival

At the end of the experiment, the seedling survival was 52% for Mix 1, 90% for Mix 2, and 80% for the control. All the surviving plants (45 for the control, 31 for Mix 1, and 56 for Mix 2) for a total of 132 plants were analyzed for mycorrhization, and morphometric assessment as reported below.

#### Percentage of truffle mycorrhization

**Fig. 1:**
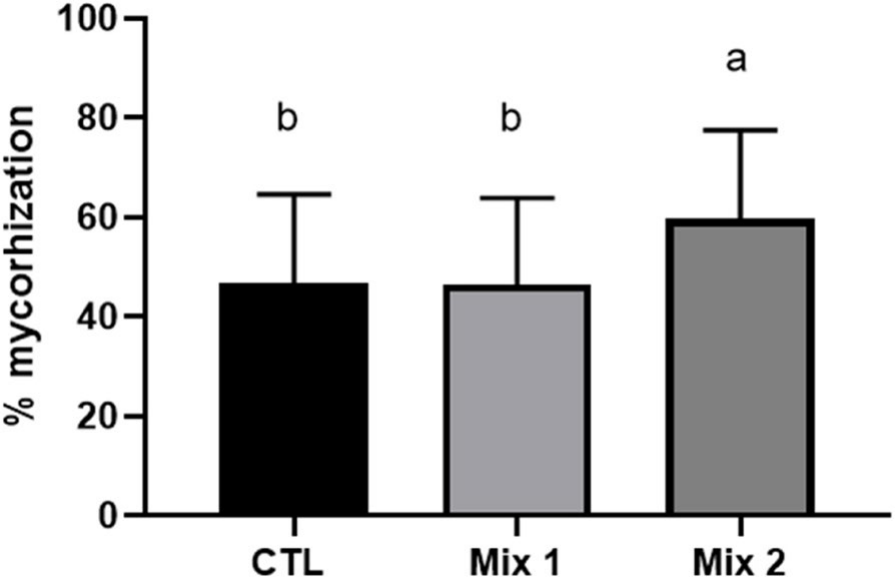
Percentages of ECM short roots were obtained by inoculation of *Q. pubescens* with *T. melanosporum*. Different letters indicate significant differences between substrates according to Tukey’s test (*p* < 0.05)

Data obtained with the RAV method (Fig. 1) showed an elevated level of mycorrhization in the control potting mix (46.7%). When growing in potting Mix 1, the percentage of ectomycorrhizal short roots was comparable to the control with percentage values of 46.4%. On the other hand, when growing on Mix 2, *Q. pubescens* seedlings resulted in a statistically higher colonization rate with 59.7% of mycorrhization (F value = 8.739, p = 0.0003). All amplified samples of mycorrhized root tips gave an estimated amplicon of ~ 500 bp, which perfectly matched the amplicon size obtained from the *T. melanosporum* ascocarp’s DNA according to Sèjalon-Delmas et al. [43]. No other ECMs morphotypes different from the inoculated species were detected, according to the different morphotypes reported by [48].

#### Shoot and root analyses

Data transformation was needed for all datasets except root dry weight. Shoot height and diameter and root length datasets were transformed with the function $Y=\sqrt{\left(X+3/8\right)}$. Shoot dry weight, number of root tips, and forks were transformed with the function $Y=\sqrt[3]X$ [49, 50]. *Q. pubescens* seedlings showed a significantly higher value of shoot diameter in potting Mix 1 (F = 8.487; *P* = 0.0003) while no differences in shoot height and dry weight were found between substrates (Table 2).

**Table 2:** Shoot development of *Q. pubescens* seedlings 8 months after inoculation (*n* = 30). Different letters indicate significant differences at the Tukey’s post-hoc test

Morphometrical parameters	Control	Mix 1	Mix 2
Shoot Diameter (mm)	1.10 b	1.43 a	1.11 b
Shoot Height (cm)	1.30 a	1.38 a	1.43 a
Shoot dry weight (g)	0.78 a	0.72 a	0.78 a

Root parameters, except root dry weight, were significantly higher on Mix 2 than on the other treatments (Table 3). Specifically, number of tips (*F* value = 13.49, *p* < 0,0001), number of forks (*F* value = 15.83, *p* < 0.0001), root length (*F* value = 17.84, *p* < 0.0001).

**Table 3:** Root development of *Q. pubescens* seedlings 8 months after mycorrhization with *T. melanosporum* in containers under controlled conditions (*n* = 30). Different letters indicate significant differences at the Tukey’s post-hoc test

Root parameters	Control	Mix 1	Mix 2
Average n. of root tips	1,929 b	1,516 b	3,329 a
Average n. of root forks	1,960 b	1,975 b	3,941 a
Total root length (cm)	349 b	272 b	531 a
Root dry weight (g)	1.99 a	1.72 a	2.16 a

**Fig. 2:**
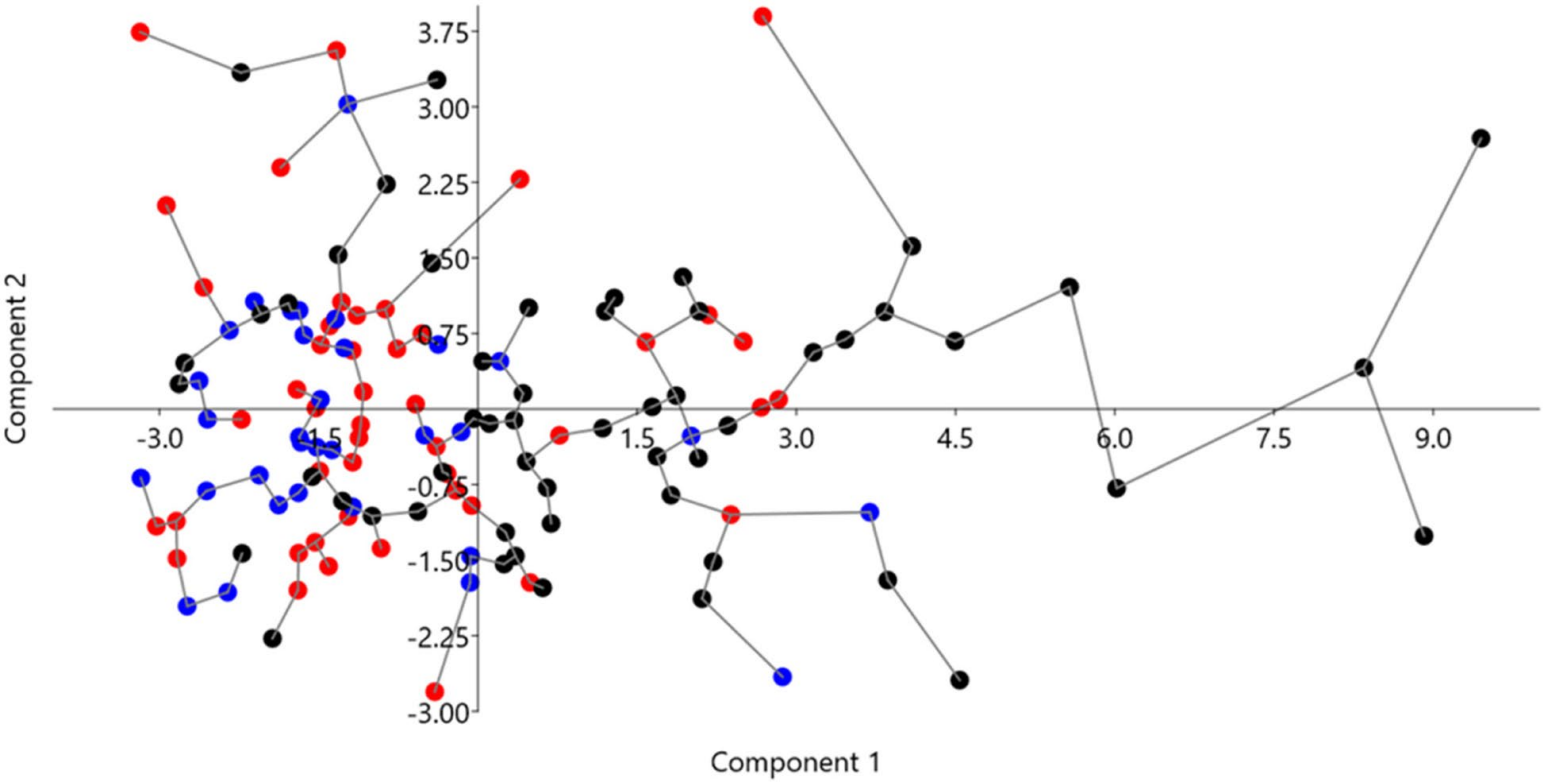
PCA analysis using shoot and root parameters, and percent of mycorrhization for each individual grown in the three potting mixes. The minimal spanning tree as the shortest possible set of lines connecting all points is also shown. Red, blue and black dots indicate control, Mix1, and Mix2 respectively

A PCA was carried out using the shoot and root parameters, and the percent of mycorrhization as descriptive variables for each potting mix. A correlation matrix was chosen because variables were measured with different units. Row-wise bootstrapping was used (1000). The results are shown in Fig. 2. Components 1 and 2 accounted for 48.2 and 14.7% of the total variance. There was not a sharp separation of groups, but, while control and Mix1 individuals appear to cluster together, there is an evident tendency of most individuals of Mix 2 to cluster separately along component 1.

## Discussion and conclusion

The present study provides the opportunity to adopt soiless substrates from a circular economy context (green, organic composts) to support the production of quality mycorrhized plants with *T. melanosporum*. Green compost based substrate provided the best performances significantly increasing the seedling survival and and mycorrhization. These results are of great interest since combine the use of a environmentally sustainable substrate with better plant performance and quality of symbiosis. Performance of plants in Mix 1 were not significantly different from the control except for shoot diameter that resulted larger. Mycorrhization and root development were satisfactory, and based on these parameters Mix 1 might be recommendable as substrate. However, the high mortality of seedlings recorded evidences a phytotoxic effect that represent an important limit for the employment in mass production of elite mycorrhized plants. Mix 1, unlike Mix 2, is coded as mixed compost amendment since utilizes municipal waste as one of the matrix. The aerobic fermentation of municipal waste might lead to an excess of phytotoxic substances that could have a negative impact on seedlings survival [51]. Commonly such phytotoxic effect decreased with maturation and storage of the final product [52], suggesting that also Mix 1 might be employeable upon a longer storage and maturation. Indeed, the results of the chemical-physical analyses of the mixed substrates used in the present study highlighted that the two compost-based amendments present high/very high mineral nutrient values. However, some consideration must be done about the presence of specific elements. The boron content of Mix 1 and 2 is very high with possible phytotoxic effects. According to [53], the requirements for boron differ significantly between plant species and genotypes within a species. Boron is a mineral element that is employed by plants in the metabolism of phenolic acids and the biosynthesis of lignin, which is directly related to the preservation of cell walls [54]. In this trial, *Q. pubescens* seedlings grown on Mix 2, characterized by an optimal pH value for black truffle and an elevated boron content, showed the highest development of the root apparatus (number of root tips and forks, and total root length) combined with a significantly higher mycorrhization rate (Table 3). This could be in line with the evidence reported by [55], regarding the positive effect of increased boron availability on ECM symbiosis. Indeed, the positive influence of increased boron availability is known on the formation of ECMs [56]. For these reasons, the presence of boron for plant growth and mycorrhizal occurrence is crucial as its deficiency is common in more than eighty countries in the world [57], especially in sandy soils and soils with an alkaline pH where this element is more easily leached [58, 59]. Moreover, in the experimental conditions, the moisture was set between 50–70% [60], so there should be no problem states of boron deficiency because in arid conditions with dry soil boron deficiencies are increased [61]. However, boron excess in plants might also have phytotoxic effects leading to chlorosis and mortality [62]. Mix 1 showed the higher content of boron and the higher mortality of seedlings, although the association in cause and effect must be considered just speculative in the present study. Regarding the sodium content, it is worth noting that the two compost-based amendments present higher sodium levels than the control one, even if not enough to be considered sodic soils [63]. In this respect, Mix 1 and Mix 2 (Table 1) present an Exchangeable Sodium Percentage (ESP) of 4.8% and 5.2%, respectively. However, this aspect did not modify the capacity of *T. melanosporum* to colonize the plants of *Q. pubescens* which showed significantly higher levels of mycorrhizal colonization in Mix 2 compared to the control. Indeed, also [64] reported that the salt tolerance of *Quercus* spp. seems to be improved by ectomycorrhizal inoculation. In addition, the alkaline pH of Mix 2 should have enhanced the mycorrhizal symbiosis relieving the sodium effect. Finally, the values of phosphorus are very high for both the compost-based substrates, but this aspect did not influence the growth and mycorrhization of the plants. Instead, the low level of phosphorus in the control could be explained by the efficiency of *T. melanosporum* in increasing its uptake, increasing the mycorrhization rate. Indeed, this tendency has been observed in Mediterranean environments where limy forest soils are typically lacking in this mineral element [65].

Furthermore, mycorrhization experiments carried out in the present study highlighted that, after spore slurry inoculation with *T. melanosporum,* ECMs were formed on all potting mixes, matching, and exceeding the quality of standards of European producers [66] In fact, it has been observed that the addition of organic matter in the form of fermented municipal compost and green organic wastes has a good impact on the ability of mycorrhizal fungi to proliferate and colonize [67]. For example, [68] used pine bark compost to evaluate the efficacy of the controlled inoculation of *Quercus cerris* and *Quercus robur* with *Tuber melanosporum*, revealing that with small pH adjustments, these substrates are particularly effective. A wide number of studies have demonstrated that mycorrhizal symbiosis enhances nutrient transport between plant symbionts and soil fungi, resulting in growth benefits for the partners [69–71]. The incorporation of the organic amendment was also reported by [72] to improve by over 20% the shoot growth of *Pinus halepensis*, and by more than 43% the volume and dry biomass. In field trials, *P. halepensis* and other plants and shrub species have achieved comparable results using different methods of soil fertilization including composted urban waste and organic additives [73, 74].

Results of PCA revealed, as expected, that root variables were those that mostly explained the differences of percent of mycorrhization. As stated above in the result section, the three groups were not distinct each other, although there was a tendency of individuals of Mix 2 to separate from control and Mix 1 ones. However still a high variability is present between individuals of Mix 2 that deserves to be reduced. An upscaling of the present study should focus on the characteristics of Mix 2 in terms balance in composition of starting matrices, quality of the fermentation process and final maturation, in order to provide standards of process and to deliver a final product specifically devoted to production of elite mycorrhizal plants. Finally, the advantages of using potting Mix 2 are several: it is a commercial product with a standardized composition stated on the label, composed of recycled substrates that are not expensive; it is environmentally sustainable; and it can offer excellent mycorrhization performances with *T. melanosporum*. In conclusion, the results reported, indicate that inoculation with *T. melanosporum* spore-slurry and the use of alternative, cheaper, and reused materials are suitable nursery practices to optimize the production of ectomycorrhizal *Q. pubescens* plants in controlled conditions.

## Supplementary Information

**Additional file 1.**

## Data Availability

The datasets used and/or analysed during the current study are available from the corresponding author on reasonable request.
